# Protein arginine methyltransferase 5 regulates multiple signaling pathways to promote lung cancer cell proliferation

**DOI:** 10.1186/s12885-016-2632-3

**Published:** 2016-08-02

**Authors:** Xiumei Sheng, Zhengxin Wang

**Affiliations:** 1 School of Medicine, Jiangsu University, Zhenjiang, Jiangsu Province 2012013 China; 2 The Center for Cancer Research and Therapeutic Development, Department of Biological Sciences, Clark Atlanta University, 223 James P. Brawley Drive, S.W, Atlanta, GA 30314 USA

**Keywords:** PRMT5, WDR77, p44, FGFR, ErbB, BTG2, GLIPR1, Lung cancer

## Abstract

**Background:**

Protein arginine methyltransferase 5 (PRMT5) catalyzes the formation of symmetrical dimethylation of arginine residues in proteins. WD repeat domain 77 (WDR77), also known as p44, MEP50, or WD45, forms a stoichiometric complex with PRMT5. The PRMT5/p44 complex is required for cellular proliferation of lung and prostate epithelial cells during earlier stages of development and is re-activated during prostate and lung tumorigenesis. The molecular mechanisms by which PRMT5 and p44 promote cellular proliferation are unknown.

**Methods:**

Expression of PRMT5 and p44 in lung and prostate cancer cells was silenced and their target genes were identified. The regulation of target genes was validated in various cancer cells during lung development and tumorigenesis. Altered expression of target genes was achieved by ectopic cDNA expression and shRNA-mediated silencing.

**Results:**

PRMT5 and p44 regulate expression of a specific set of genes encoding growth and anti-growth factors, including receptor tyrosine kinases and antiproliferative proteins. Genes whose expression was suppressed by PRMT5 and p44 encoded anti-growth factors and inhibited cell growth when ectopically expressed. In contrast, genes whose expression was enhanced by PRMT5 and p44 encoded growth factors and increased cell growth when expressed. Altered expression of target genes is associated with re-activation of PRMT5 and p44 during lung tumorigenesis.

**Conclusions:**

Our data provide the molecular basis by which PRMT5 and p44 regulate cell growth and lay a foundation for further investigation of their role in lung tumor initiation.

**Electronic supplementary material:**

The online version of this article (doi:10.1186/s12885-016-2632-3) contains supplementary material, which is available to authorized users.

## Background

Protein arginine methyltransferase 5 (PRMT5) is a type II protein arginine methyltransferase that catalyzes the symmetrical dimethylation of arginine residues within target proteins and has been implicated in diverse cellular and biological processes including transcriptional regulation [1–3], RNA metabolism [4, 5], ribosome biogenesis [6], Golgi apparatus structural maintenance [7], and cell cycle regulation [1]. In mammalian cells, PRMT5 localizes to both the cytoplasm and the nucleus and it methylates multiple histone and nonhistone proteins [4]. In the nucleus, PRMT5 has been found in the SWI/SNF and NuRD chromatin-remodeling complexes [8, 9], where it can methylate histones as well as transcription factors or regulators [1–3]. In the cytoplasm, PRMT5 forms a 20S protein arginine methyltransferase complex termed the “methylosome”, consisting of spliceosomal snRNP Sm proteins, PRMT5, pICln, and WD repeat protein (MEP50) to function as a master regulator of splicing [10–12].

PRMT5 directly methylates epidermal growth factor receptor (EGFR), E2F1, and p53 to promote cell survival and growth [1, 13–15]. Cyclin D/Cdk4 kinase enhances PRMT5 activity to trigger neoplastic growth [16]. Inhibition of PRMT5 activity redirected the response of PC12 cells to EGF from proliferation to differentiation [17]. Given these roles, PRMT5 is generally regarded to promote tumor growth. In agreement with this conclusion, PRMT5 has been found over-expressed in leukemia, lymphoma, colorectal, lung, ovarian, and prostate cancer [14, 18–22]. A selective inhibitor of PRMT5 demonstrated antitumor activity in multiple mantle cell lymphoma xenograft models [23]. PRMT5 and p44 in the cytoplasm are required for proliferation of prostate epithelial cells [21, 24–26]. In contrast, PRMT5 and p44 in the nucleus in function with the androgen receptor to drive prostate epithelial cell differentiation and function [24, 27, 22]. Translocation of PRMT5 and p44 from the nucleus to the cytoplasm is associated with prostate tumorigenesis demonstrating that cytoplasmtic PRMT5 and p44 are required for growth of prostate cancer [21, 25, 27]. More recently, we found that p44 is highly expressed in the lung’s earlier stages of development when cells are rapidly proliferating whereas its expression is diminished in adult lungs when cells are fully differentiated [28]. Loss of the *p44* gene led to growth arrest and differentiation of lung epithelial cells. More important, PRMT5 and p44 are re-expressed in lung cancers and the shRNA-mediated silencing of PRMT5 or p44 expression strongly inhibited proliferation of lung cancer cells in tissue culture and abolished growth of lung tumor xenografts in nude mice [20, 28]. These results reveal a novel role of PRMT5 and p44 in growth of lung and prostate epithelial cells as well as lung and prostate cancers.

In searching for molecules that mediate PRMT5/p44 functions in cell growth, we performed DNA microarray analysis with lung adenocarcinoma A549 cells expressing PRMT5 or p44 shRNA and identified a set of genes targeted by both PRMT5 and p44. Altered expression of these genes was observed during mouse lung development and lung tumorigenesis and affected growth of lung cancer cells. Our results demonstrate PRMT5 and p44 regulation of gene expression of growth and anti-growth factors to promote cell growth.

## Methods

### Cell culture and growth assay

A549 and PC14 cells were cultured in minimum essential medium (CellGro) with 10 % (v/v) fetal bovine serum (FBS) (HyClone), 2 % vitamins, 1 % L-glutamine, 1 % non-essential amino acids, and 1 % sodium pyruvate. PC3 and LNCaP cells were cultured in RPMI 1640 medium (CellGro) with 10 % FBS. For cell growth assays, cells were plated on 24-well plates (2,000 cells/well) and counted 6 days later. For bromodeoxyuridine (BrdU) (BD Biosciences) incorporation assays, cells (50–70 % confluence) were plated on a chamber slide (BD falcon) and cultured in the presence of 10 μM BrdU for 4 h. The BrdU-positive cells were detected by immunostaining with the monoclonal anti-BrdU antibody (BD Biosciences) as described previously [24, 28].

### Lung samples and immunohistochemical staining

Lung tumor samples were obtained from existing pathological specimens at Tangdu Hospital (Xi’an, China), and the study protocol was approved by its institutional review board [28]. BALB/c mice were purchased from the National Cancer Institute and maintained in a barred animal facility. The lungs of the mice were removed and fixed with formaldehyde [29]. Mice were handled in accordance with the guidelines published in the National Institutes of Health Guide for the Care and Use of Laboratory Animals. The Morehouse College School of Medicine’s Institutional Animal Care and Use Committee approved all the experimental procedures used for mice in this study. Antigen retrieval and immunostaining were performed as described previously [29, 21]. Briefly, formalin-fixed, paraffin-embedded tissue sections were deparaffinized by sequential washing with xylene, graded ethanol, and phosphate-buffered saline (PBS). Antigen retrieval was done by heating the samples in a steam cooker in citrate buffer (pH 6.0) for 30 min. After the samples were cooled and washed with PBS, endogenous peroxide was blocked with 3 % hydrogen peroxidase inhibitor in PBS for 12 min. Nonspecific proteins were blocked by immersing the sections in 5 % horse serum and 1 % goat serum for 20 min. Slides were incubated with primary antibodies overnight at 4 °C and then with a secondary peroxidase-labeled anti-rabbit antibody (1:500; Jackson ImmunoResearch) for 1 h at room temperature. Signal was detected by staining with 3, 3′-diaminobenzidine (DAB) (Phoenix Biotechnologies) substrate for 6 min and then counterstaining with Gill’s Hematoxylin No. 3 (Sigma) for 20 s. Immunostaining without the primary antibody served as a negative control.

### RNA interference and gene expression profiling

The non-target (NT), p44, and PRMT5 small hairpin RNA (shRNA) and lentivirus production were described previously [20, 28]. Briefly, A549 cells were plated at 60 % confluency in 6-well plates and transduced with the lentivirus. After 16 h, the virus-containing medium was removed and replaced with a normal growth medium. Three days after infection, cells were split at 1:6 and allowed to grow for 3 days. Whole-cell lysates and total RNAs were prepared and subjected to Western blot analysis and real-time polymerase chain reaction. Cell growth assays and BrdU incorporation assays were performed as described (above). A gene expression profiling analysis was performed on A549 cells expressing p44 shRNA, PRMT5 shRNA, or NT shRNA. Total RNA was extracted from cells using the RNAqueous Total RNA Isolation kit (Ambion, Austin, TX) 4 days post virus infection. After confirmation of RNA quality using a Bioanalyzer 2100 instrument (Agilent), 300 ng of total RNA was amplified and biotin-labeled through an Eberwine procedure using an Illumina TotalPrep RNA Amplification kit (Ambion) and hybridized to Illumina HT12 version 4 human whole-genome arrays. Processing of bead-level data was by methods previously described [30]. Significance testing for differentially-expressed probes was by the Wilcoxon Rank-Sum Test applied to individual processed bead values, with false-discovery rate significance values (q) determined by the method of Benjamini and Hochberg [31]. Hierarchical clustering and heat mapping used Cluster and Treeview software from Eisen et al. [32]. Gene set analysis applied gene set enrichment analysis (GSEA) [33] and the hypergeometric distribution test [34] to gene sets from mSigDB and individual literature sources. The gene expression data was analyzed using R Program (www.r-project.org) and comparison was used to select genes differentially expressed at a significance level of *p* < 0.001. Microarray data presented in this study has been deposited in the GEO public repository (GSE56757).

### Ectopic gene expression

Human cDNA clones encoding GLI Pathogenesis-Related 1 (GLIPR1), leucine proline-enriched proteoglycan (Leprel1), B-cell translocation gene 2 (BTG2), and V-Erb-B avian erythroblastic leukemia viral oncogene homolog 3 (ErbB3) were purchased from DNASU and subcloned into the lentiviral expression vector (pCDH, System Biosciences). Lentivirus was prepared as described previously [29]. Cells (4 ×10^4^) were plated in 6-well plates and transduced with lentivirus. After 48 h, cells were replated in a 100-mm dish, and 2 days later protein expression was confirmed by Western blot analysis.

### Real-time PCR

Total RNAs were isolated from cultured cells or mouse lung tissues using TRIzol reagent and reverse transcribed into cDNA using the ReactionReady First Strand cDNA Synthesis Kit (SuperArray Biosciences). The cDNA products were PCR-amplified with the RT2 Real-Time SYBR Green qPCR master mix (SuperArray Biosciences) and the gene-specific primer sets (Additional file 1: Table S1) using a SmartCycler II (Cepheid; 40 cycles of 30 s at 94 °C, 30 s at 55 °C and 30 s at 72 °C). The SmartCycler software program (version 2.0 C) was used to process and quantify raw data. The 2^*-ΔΔCT*^ method was used to relatively quantify target gene expression as described previously [35].

### Western blot analysis

Protein extracts made from cultured cells were subjected to 10 % sodium dodecyl sulfate-polyacrylamide gel electrophoresis and then transferred to Immobilon-P membranes (Millipore). The membranes were washed in Tris-buffered saline with Tween 20 (10 mM Tris-HCl, pH 8, 150 mM NaCl, 0.05 % Tween 20) and blocked with 3 % nonfat milk in Tris-buffered saline with Tween 20 for 1 h. The blots were then probed for 2 h with the primary antibody at dilutions of 1:1,000 (anti-p44), 1:1,000 (anti-FGFR3), or 1:5,000 (anti-β-actin, Sigma-Aldrich) or overnight with the primary antibody at dilutions of 1:1,000 (anti-ErbB3) or 1:1,000 (anti-GLIPR1). The blots were then incubated with a horseradish peroxidase-conjugated secondary antibody for 1.5 h. Immunoreactive proteins were detected using an enhanced chemiluminescence detection system (GE Healthcare) per the manufacturer’s instructions. Protein concentrations were determined using the Bradford protein assay (Bio-Rad). The protein bands were scanned using a densitometer and the relative intensities were quantified using the ImageJ software program (ImageJ64, National Institutes of Health).

### Fluorescence-activated cell sorting (FACS) analysis

Cells (50–60 % confluency) were harvested, washed with PBS, and fixed in 70 % ethanol at 4 ^o^C overnight. Cells were collected and stained with propidium iodide (PI). The cell-cycle distributions were determined by flow cytometry analysis as described [28].

### Terminal deoxynucleotidyl transferase dUTP nick end labeling (TUNEL)

The TUNEL assay was performed with cells cultured in chamber slides using the apoptosis detection kit (Promega) according to the manufacturer’s instructions. Slides were evaluated under a microscope (Olympus IX71) with a digital camera (Retiga 1300) interfaced to a computer with PCI software. The apoptotic cells were quantified as follows. The number of cells of the area (DAPI staining) were captured and counted. There were approximately 100 cells per area and three areas were captured in each slide. The fluorescence-labeled cells (apoptotic cells) from the same area were captured and counted. Three independent experiments were performed and analyzed.

### Statistical analysis

Data are presented as the means of three independent experiments ± the standard deviation. A 2-tailed unpaired student *t*-test was used to determine whether differences between control and experiment samples were statistically significant. *P* values less than 0.05 were considered statistically significant. *, *p* < 0.05; **, *p* < 0.01; ***, *p* < 0.001.

## Results

### Cellular proliferation requires both PRMT5 and p44

**Fig. 1:**
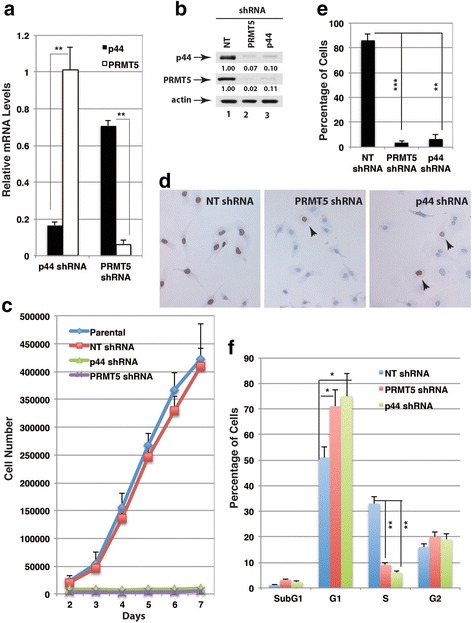
Silencing PRMT5 and p44 expression suppresses cellular proliferation and arrests cell cycle at the G1 phase. **a**, **b** Silencing p44 or PRMT5 expression in A549 cells. DNA microarray analysis of PRMT5 and p44 mRNA (**a**) or Western blot of p44 and PRMT5 protein levels (**b**) in A549 cells expressing NT, p44, or PRMT5 shRNA. Relative mRNA or protein levels = signals in cells expressing target shRNA/signals in cells expressing NT shRNA. **c** Silencing p44 or PRMT5 inhibited cell growth. Growth curves of parent A549 cells or A549 cells expressing NT shRNA, p44 shRNA, or PRMT5 shRNA. **d** Silencing p44 or PRMT5 suppressed cellular proliferation. A549 cells expressing NT shRNA, p44 shRNA, or PRMT5 shRNA were allowed to grow in the presence of BrdU and immunostained with the anti-BrdU antibody (brown). The black arrows (right two panels) indicate the BrdU-positive stained cells. **d** Percentage of BrdU-positive cells in A549 cells infected with NT, PRMT5, or p44 shRNA-expressing lentivirus. **e** Percentage of BrdU-positive A549 cells that expess NT, p44, or PRMT5 shRNA. **f** Silencing p44 or PRMT5 arrested cell cycle at the G1 phase. The cell-cycle distribution in A549 cells expressing NT shRNA, p44 shRNA, or PRMT5 shRNA by using flow cytometric analysis

Infection of lung cancer A549 cells with lentivirus expressing PRMT5 or p44 small hairpin RNA (shRNA) resulted in 94 % or 83 % reduction in PRMT5 or p44 mRNA levels, respectively (Fig. 1a). The shRNA specificity has been tested by the rescue experiments previously [20, 28]. PRMT5 shRNA expression dramatically decreased both PRMT5 (98 % reduction) and p44 (93 % reduction) protein levels (Fig. 1b, lane 2). Similarly, p44 shRNA expression significantly decreased both p44 (90 % reduction) and PRMT5 (89 % reduction) protein levels (Fig. 1b, lane 3). However, silencing p44 expression did not affect PRMT5 mRNA expression and silencing PRMT5 slightly decreased (30 % reduction) p44 mRNA expression (Fig. 1a), which is statistically significant (*p* = 0.01755). These results are consistent with previous findings that p44 and PRMT5 proteins form a stoichiometric complex and co-exist in the cytoplasm [21].

Silencing PRMT5 or p44 expression almost completely abolished cell growth (Fig. 1c). We then performed the bromodeoxyuridine (BrdU) incorporation assay to measure cell proliferation. Cells that had BrdU incorporation (i.e., cells in S-phase) were identified by immunohistochemistry with an anti-BrdU antibody. The majority (86 %) of NT shRNA-expressing cells were labled with BrdU (Fig. 1d, left panel; Fig. 1e). Silencing PRMT5 or p44 expression dramatically decreased the cellular proliferation rate (PRMT5: 96.8 % reduction; p44: 93.5 % reduction) (Fig. 1d, middle and right panels; Fig. 1e). Fluorescence-activated cell sorting (FACS) analysis indicated that silencing PRMT5 or p44 resulted in cell cycle arrest at the G1 phase (Fig. 1f). Silencing p44 also led to slight G2 arrest that is not statistically significant. It was shown that PRMT5 regulated apoptosis in some cancer cells [36, 37]. However, silencing PRMT5 or p44 did not significantly affect apoptosis in A549 cells (Fig. 1f, subG1 population; Additional file 2: Figure S1, TUNEL assay). Thus, PRMT5 and p44 are required for lung cancer cell proliferation.

### Identification of genes targeted by PRMT5 and p44

A gene expression profiling analysis was performed on A549 cells expressing p44 shRNA, PRMT5 shRNA or NT shRNA (GSE56757). Gene set enrichment analysis (GSEA) indicates that genes up-regulated by PRMT5 shRNA were over-represented on the gene list, whose expression is negatively associated with cell proliferation (Additional file 3: Figure S2). Consistent with this observation, a GSEA enrichment plot indicates that genes up-regulated by PRMT5 shRNA were over-represented on the gene list whose expression is negatively associated with cell cycle progression (Additional file 4: Figure S3). These results further confirm the PRMT5’s role in cellular proliferation.

**Fig. 2:**
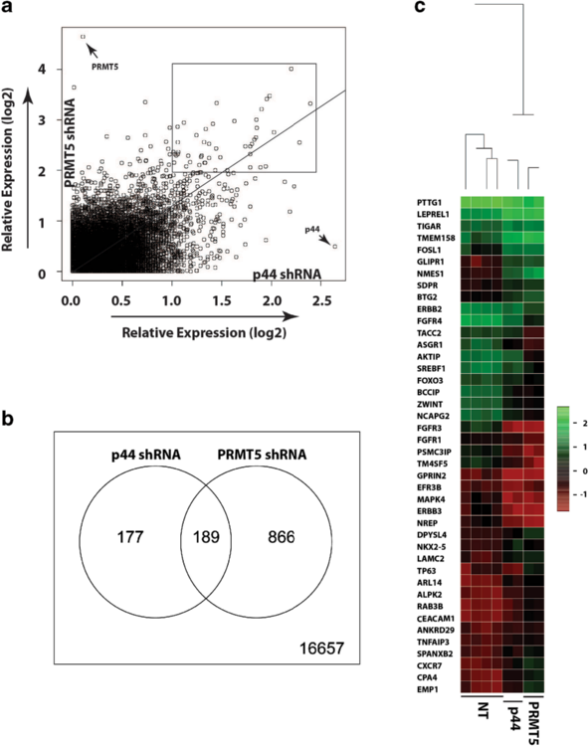
Silencing p44 or PRMT5 expression causes transcriptional deregulation of a subset of genes. **a** Scatter plots were performed to compare global gene expression profiles in p44- and PRMT5-silencing A549 cells. Genes whose expression altered more than 2-fold in p44 and 4-fold in PRMT5 expressing cells are circled by the rectangular box. **b** Venn diagram shows genes whose expression altered more than 2-fold in p44 or PRMT5 shRNA expressing cells compared to NT shRNA expressing cells. **c** Genes targeted by both p44 (>2-fold) and PRMT5 (>4-fold) are visualized by heatmap. The *blue*, *black*, and *red colors* represent higher than average, close to average, and lower than average expression of particular gene, respectively. The rows are organized by hierarchical clustering using agglomerative clustering with complete linkage and Euclidian distance metric

**Fig. 3:**
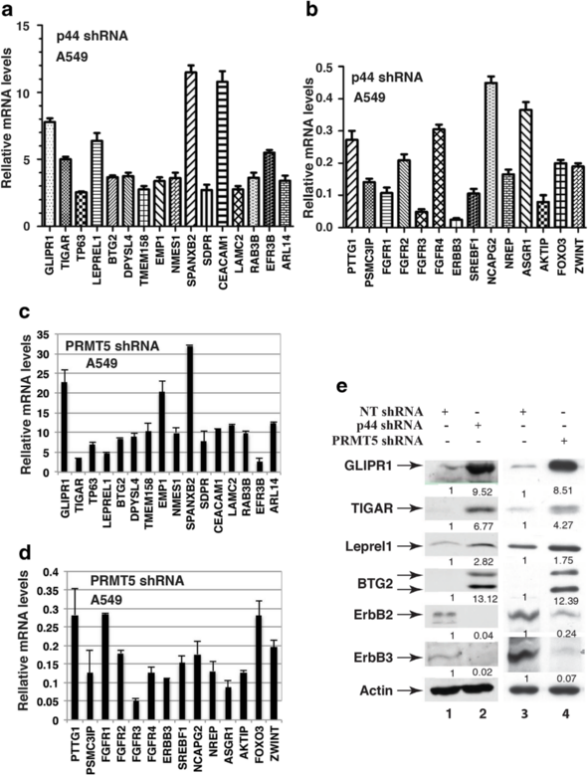
Identification of genes whose expression altered in p44 or PRMT5 silencing cells. **a-d** RT-PCR analysis of gene expression. Relative mRNA levels = mRNA in A549 cells expressing p44 (**a**, **b**) or PRMT5 (**c**, **d**) shRNA/mRNA in NT shRNA expressing A549 cells. **e** Western blot analysis of whole-cell lysates derived from NT (lanes 1 and 3), p44 (lane 2), or PRMT5 (lane 4) shRNA-expressing A549 cells with antibodies as indicated

Scatter plots were performed to compare global gene expression profiles in PRMT5- and p44-silencing A549 cells (Fig. 2a). There are 189 genes regulated by both PRMT5 and p44, 866 genes regulated by PRMT5 only and 177 genes regulated by p44 only (>2-fold and *p* < 0.001) (Fig. 2b). We choose 42 genes whose expression was regulated by both PRMT5 (>4-fold) and p44 (> 2-fold) for further analysis (Fig. 2a, surrounded by the rectangle) since PRMT5 shRNA was approximately 2-fold more effective than p44 shRNA (Fig. 1a). The gene heatmap reports changes in expression of these genes (Fig. 2c). Among these genes, 23 genes are down regulated and 19 genes are up regulated by PRMT5 and p44. RT-PCR confirmed the regulation of 20 genes by p44 (Fig. 3a, b) and PRMT5 (Fig. 3c, d) in A549 cells. We failed to obtain specific RT-PCR products for the other 12 genes due to their low gene expression profiles or improper RT-PCR conditions.

**Fig. 4:**
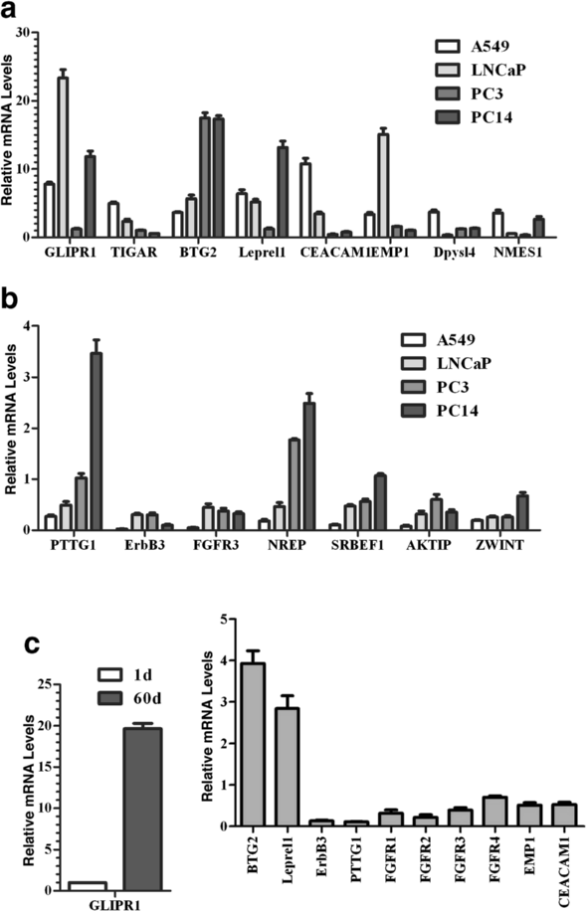
Expression of p44/PRMT5-target genes in cancer cell lines and in mouse lungs. **a**, **b** RT-RCR analysis of gene expression in cancer cell lines. Relative mRNA levels = mRNA in cells expressing p44 shRNA/mRNA in NT shRNA expressing cells. **c** RT-PCR analysis of gene expression in mouse lungs. RNAs were isolated from lung tissues obtained from mice at the age of 1 or 60 days. GLIPR1-expressing cells are stained in brown. Relative mRNA levels = mRNA from lungs of 60-days old mice/mRNA from lungs of 1-day old mice

These analyses were extended to the other lung cancer (PC14) and two prostate cancer (PC3 and LNCaP) cell lines. PC3 is an androgen-independent cancer cell line and LNCaP is an androgen-dependent cancer cell line, which are broadly used in prostate cancer research [38]. Similarly to A549 cells (Fig. 1), silencing p44 or PRMT5 in these cells also suppressed cellular proliferation [20, 21, 24, 28, 39]. B-cell translocation gene 2 (BTG2) gene expression was up regulated and expression of V-Erb-B avian erythroblastic leukemia viral oncogene homolog 3 (ErbB3) and fibroblast growth factor receptor 3 (FGFR3) genes were down-regulated in all 4 cell lines when p44 expression was silenced (Fig. 4a, b). Expression of the other genes was variable among the 4 cell lines in response to p44 silencing. For example, expression of GLI pathogenesis-related 1 (GLIPR1) and leucine proline-enriched proteoglycan (Leprel1) genes was induced by p44 silencing in A549, LNCaP and PC14 cells but not in PC3 cells (Fig. 4a).

In our previous study [32], we reported that p44 was highly expressed in the lungs of mice at the ages of 1–14 days, while absent in the lungs of mice at the ages of 60–390 days. We performed RT-PCR analysis to detect expression of identified PRMT5/p44 target genes. Expression of GLIPR1, BTG2, and Leprel1 genes increased whereas expression of PTTG1, ErbB3, FGFR1/2/3/4, epithelial membrane protein 1 (EMP1), and carcinoembryonic antigen-related cell adhesion molecule 1 (CEACAM1) genes decreased in fully developed murine lungs (60 days) compared with proliferating murine lungs (1 day) (Fig. 4c).

Based on these analyses, we performed Western blot analysis (Fig. 3e) for proteins of 6 genes whose mRNA expression response to PRMT5/p44 demonstrated the same trend in most (3 out of 4), if not all, cancer cell lines as well as during lung development. The protein levels of these 6 genes were altered in the same trend as observed in the RT-PCR analysis in response to silencing p44 (Fig. 3e, lanes 1 and 2) or PRMT5 (lanes 3 and 4).

### Ectopic expression of PRMT5/p44-suppressed genes inhibits cell growth

**Fig. 5:**
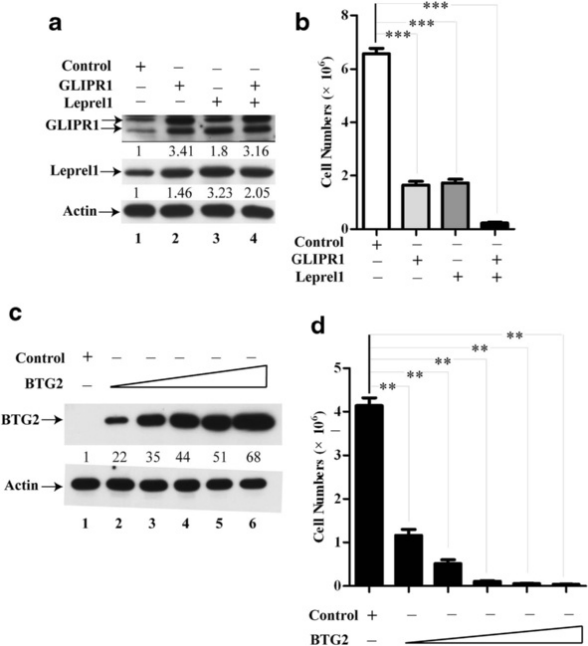
Ectopic expression of p44/PRMT5-target genes inhibits cell growth. **a** Western blot analysis of whole-cell lysates derived from A549 cells infected with the control lentivirus (lane 1) or lentivirus expressing GLIPR1 (lane 2), Leprel1 (lane 3) or both GLIPR1 and Leprel1 (lane 4). **b** Growth of A549 cells infected with control lentivirus or lentivirus expressing GLIPR1, Leprel1 or both. **c** Western blot analysis of whole-cell lysates derived from A549 cells infected with the control lentivirus (lane 1) or lentivirus expressing BTG2 (lanes 2-6). **d** Growth of A549 cells infected with control lentivirus or lentivirus expressing BTG2

Decreased expression of *GLIPR1* gene is associated with prostate cancer and Leprel1 functions as a suppressor of cell proliferation and their down-regulation or silencing was observed in cancers [40–43]. *BTG2* gene encodes an anti-proliferation protein [44]. Silencing p44 or PRMT5 expression resulted in up-regulation of mRNA expression of *GLIPR1*, *Leprel1*, and *BTG2* genes (Fig. 2c, Fig. 3a-d). Western blot showed their protein levels were also elevated when p44 or PRMT5 was silenced (Fig. 3e). To examine the functional relevance of their expression, we ectopically expressed these proteins in A549 cells. Over expression of GLIPR1 (3.4-fold, Fig. 5a, top panel, lane 2) or Leprel1 (3.2-fold, Fig. 5a, middle panel, lane 3) significantly (4-fold) inhibited cell growth (Fig. 5b). Co-expression of GLIPR1 (3.1-fold) and Leprel1 (2.0-fold) (Fig. 5a, lane 4) shows a synergistic (20-fold) inhibitory effect on cell growth (Fig. 5b). Similarly, ectopic expression of BTG2 (Fig. 5c) led to inhibition (up to 84-fold) of cell growth in dosage dependent manner (Fig. 5d). Thus, expression of GLIPR1, Leprel1, and BTG2 induced by p44 or PRMT5 silencing could result in significant inhibition of cell growth.

### Ectopic expression of PRMT5/p44-upregulated genes enhances cell growth

**Fig. 6:**
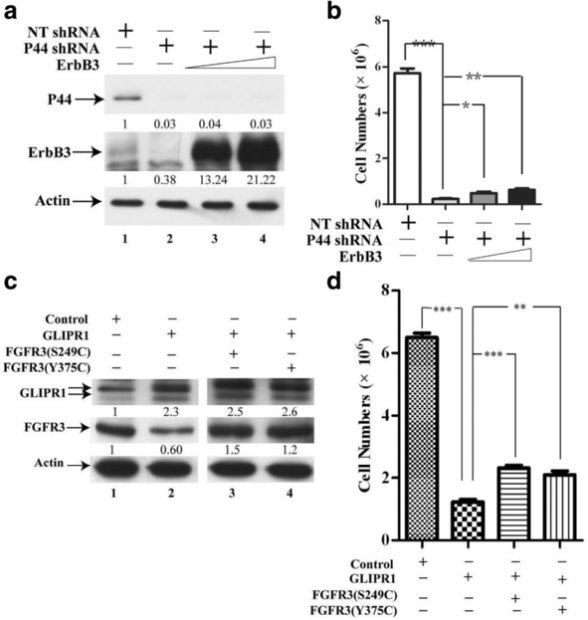
Ectopic expression of p44/PRMT5-target genes partially restores growth defect of p44-silencing cells. **a** Western blot analysis of whole-cell lysates of A549 cells expressing NT (lane 1) or p44 shRNA (lanes 2-4) infected with lentivirus expressing ErbB3 (lanes 3 and 4). **b** Expression of ErbB3 increased growth of p44-silenced cells. **c** Western blot analysis of whole-cell lysates derived from A549 cells expressing GLIPR1, FGFR3 or the combination. **d** Growth of A549 cells expressing GLIPR1 or GLIPR1 plus FGFR3

Expression of some growth factors (ErbB2/3, FGFR1/2/3/4) was significantly decreased in PRMT5 or p44 silencing cells (Figs. 2, 3 and 4). We previously showed that ectopic expression of wild type FGFR3 or constitutively activated forms [FGFR3(S249C) and FGFR3 (Y375C)] of FGFR3 partially restored growth inhibition induced by PRMT5 silencing [20]. Similarly, ectopic expression of ErbB3 slightly (statistically significantly) enhanced cell growth of p44 shRNA-expressing cells by 2.1- and 2.8-fold (Fig. 6a, b). Growth inhibition induced by GLIPR1 expression could be partially reverted by ectopic expression of ErbB3 [45] or constitutively activated forms FGFR3 [FGFR3(S249C) and FGFR3(Y375C)] (Fig. 6c, d). Thus, PRMT5 and p44 control cell growth by altering expression of these growth factors and growth suppressors.

### Expression of PRMT5/p44-target genes during lung tumorigenesis

**Fig. 7:**
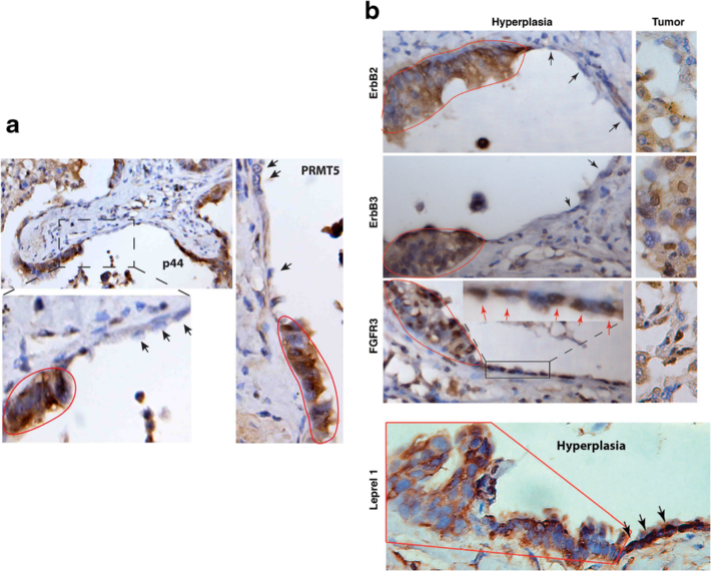
Expression of PRMT5, p44 and their target genes during lung tumorigenesis. **a** Immunostaining of p44 and PRMT5 in lung hyperplasia. The hyperplastic regions are surrounded by red lines. PRMT5- or p44-expressing cells are stained in brown. Benign epithelial cells are indicated by lack arrows. **b** Immunostaining of ERBB2/3, FGFR3 and Leprel1 in lung hyperplasia (*left*) or cancer (*right*)

PRMT5 and p44 are re-expressed in lung cancer and are required for lung tumor growth [20, 28]. The reactivation of PRMT5 and p44 was also observed in lung epithelial hyperplasia (Fig. 7a, encircled areas by red lines) whereas the nearby benign lung cells (Fig. 7a, indicated by black arrows) did not express PRMT5 or p44. Expression of PRMT5 and p44 was also observed in other types of lung hyperplasias (Additional files 5 and 6: Figures S4 and S5). These observations are consistent with previous findings that PRMT5 and p44 are required for cell proliferation [20, 28].

Similarly, ErbB2 and ErbB3 were not expressed in the benign lung epithelial cells (Fig. 7b, left top two panels, indicated by black arrows) but highly expressed in lung hyperplasia (Fig. 7b, left, circled by red lines) and in most lung cancer cells (Fig. 7b, right; Additional file 7: Figure S6). FGFR3 was expressed at low levels in benign lung epithelial cells (Fig. 7b, left, indicated by red arrows) and its expression was significantly enhanced in lung hyperplasia (Fig. 7b, left, circled by red line) and in most lung cancer cells (Fig. 7b, right; Additional file 7: Figure S6). In contrast, expression of Leprel11 was high in the nuclei of benign lung epithelial cells (Fig. 7b, indicated by black arrows) but lower in the nuclei of cells in lung hyperplasia (Fig. 7b, circled by red line). Therefor, there is a good correlation between PRMT5/p44 re-expression with expression of their target genes during lung tumorigenesis. Expression of PRMT5/p44 is ubiquitous in lung cancer [20, 28] and hyperplasia (Fig. 7a; Additional files 5 and 6: Figures S4 and S5). However, the heterogeneity in ErbB2/3 and FGFR3 expression was observed in lung cancer cells (Fig. 7b, left; Additional file 7: Figure S6), suggesting regulation of their expression by PRMT5/p44 may be lost in some cancer cells and their functions may be redundant in cancer cell growth. These expression patterns were consistent with the RNA-Seq data obtained from 1,124 lung cancer patients. The healthy normal lung tissues express low levels of PRMT5, WDR77, ErbB2, ErbB3 and FGFR3 and high levels of GLIPR1, Leprel1 and BTG2 (Additional file 8: Figure S7). In contrast, lung cancer samples express high levels of PRMT5, WDR77, ErbB2, ErbB3 and FGFR3 and decreased levels of GLIPR1, Leprel1 and BTG2 (Additional file 8: Figure S7).

## Discussion

We previous showed an essential role of PRMT5 and p44 in growth of lung and prostate cancer cells [22, 28, 21, 25]. In this study, we identified a special set of genes whose expression is regulated by both PRMT5 and p44 and demonstrated that PRMT5/p44 regulates cell growth by altering expression of their target genes.

DNA microarray assays identified a set of genes whose expression altered when PRMT5 or p44 expression was silenced. RT-PCR and Western blot analyses confirmed these observations. Regulation of identified genes by PRMT5 and p44 was further demonstrated in multiple lung and prostate cancer cell lines and in mouse lungs as well as during lung tumorigenesis. Among this set of genes, three genes (*GLIPR1*, *Leprel1*, and *BTG2*) encode proteins functioning as growth suppressors [40, 43, 44]. Their expression is frequently down regulated or silenced in cancers [42, 44, 46]. We found that silencing PRMT5 or p44 significantly induced expression of GLIPR1, Leprel1, and BTG2 at both mRNA and protein levels, indicating that PRMT5/p44 suppresses their expression. Their up-regulation also had functional consequence on cells since ectopic expression of any of these proteins strongly inhibited cell growth. Thus, PRMT5/p44 promotes cell growth partly through suppressing expression of these growth suppressors.

Silencing PRMT5 or p44 inhibited expression of genes encoding several growth factors (FGFR1/2/3/4 and ErbB2/3), indicating that PRMT5 and p44 positive regulate their expression. This regulation was observed in cancer cell lines and mouse lungs as well as in lung hyperplasia and lung cancer. We previously demonstrated that ectopic expression of FGFR3 partially restored the cell growth defect induced by silencing PRMT5 expression [20]. Similar results were observed with ErbB3. Therefore, PRMT5/p44 promotes cell growth partly through promoting expression of these growth factors. Moreover, over-expression of growth factors (ErbB3 and FGFR3) could partially overcome cell growth inhibition induced by expression of growth suppressors (GLIPR1 and BTG2). Thus, PRMT5 and p44 promote cell growth via enhancing expression of growth factors (FGFR and ErbB) while simultaneously, decreasing expression of growth suppressors (GLIPR1, BTG2, and Leprel1).

**Fig. 8:**
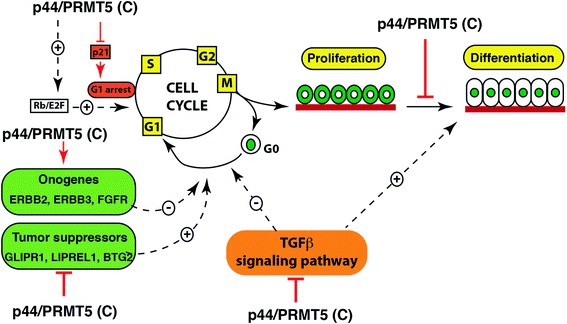
A model deciphers molecular mechanisms by which PRMT5/p44 controls cell growth. PRMT5/p44 promotes cell cycle progression from G1 to S phase by increasing Rb phosphorylation and inhibiting p21 expression. PRMT5/p44 alters expression of oncogenes and tumor suppressors, which initiates cell growth. PRMT5/p44 also regulates cell growth and differentiation through the TGFβ signaling pathway

It is noticed that either ErbB3 or FGFR3, even ErbB3 together with FGFR3, failed to fully restore the cell growth inhibition induced by p44 silencing. ErbB2 and ErbB3 are two members of the EGF receptor family [47, 48]. ErbB2 lacks ligand-binding ability, whereas ErbB3 is unique in that it does not have any intrinsic kinase activity [49, 50]. ErbB2 and ErbB3 form a heterodimer and perform a central role in maintenance and malignancy of lung and other cancers, through activating the phosphatidylinositol-4,5-bisphosphate 3-kinase (PI3K) pathway [51–55]. Combined blockade of ErbB2 and ErbB3 inhibited PI3K activity more effectively than each inhibitor alone [52, 55]. Consistent with these observations, co-expression of ErbB2 and ErbB3 was evident in some lung cancer samples (Additional file 8: Figure S7). The next experiment would co-express both ErbB2 and ErbB3 in order to better restore the growth inhibition induced by p44 silencing. It is also possible that other signaling pathways may also be regulated by PRMT5/p44. Indeed, we found that PRMT5/p44 modulates cell cycle progression from the G1 phase to the S phase by regulating p21 expression and Rb phosphorylation [28]. It was also reported that PRMT5 regulated extracellular signal-regulated kinase (ERK), E2F1, and other signaling pathways to modulate cell growth [15, 17, 39, 56, 15]. Thus, p44 and PRMT5 regulate cell growth through multiple signaling pathways including growth factors, growth suppressors, regulatory proteins in cell cycle progression, and the transforming growth factor beta (TGFβ) signaling (Fig. 8). The exact mechanisms by which PRMT5/p44 regulates expression of identified genes remain unclear. Given the fact that PRMT5/p44 has been implicated in diverse cellular and biological processes [4], this may be through regulating the splicing of transcription factors or directly methylating them, which in turn affects expression of identified genes. Further analysis is necessary to identify such transcriptional factors.

PRMT5 and p44 are highly expressed in prostate and lung cancers and are essential for growth of cancer cells [29, 31, 33, 50]. There is a good correlation between expression of PRMT5/p44-target genes with PRMT5/p44 re-expression during lung tumorigenesis, especially at the stage of hyperplasia. Although PRMT5 and p44 are ubiquitously expressed in all cancer cells [29, 31, 33, 50], we observed heterogeneous expression of ErbB2, ErbB3, and FGFR3 in lung cancer cells and of some PRMT5/p44-target genes in various cancer cell lines in response to p44 silencing. These observations may reflect the fact that they are functionally redundant in the control of cancer cell growth. Consistently with this conclusion, the RNA-Seq data demonstrated that lung cancer samples express high levels of ErbB2 and ErbB3 or FGFR3 (Additional file 8: Figure S7).

## Conclusion

This study is the first to identify specific transcriptional programs associated with PRMT5 and p44. Our data provides critical downstream cellular factors/pathways and potential molecular mechanisms of PRMT5/p44-driving cellular proliferation.

## Abbreviations

BrbB, V-Erb-B avian erythroblastic leukemia viral oncogene homolog; BrdU, bromodeoxyuridine; BTG2, B-cell translocation gene 2; CEACAM1, carcinoembryonic antigen-related cell adhesion molecule 1; DAB, 3, 3′-diaminobenzidine; EMP1, epithelial membrane protein 1; ERK, extracellular signal-regulated kinase; FACS, fluorescence-activated cell sorting; FBS, fetal bovine serum; FGFR, fibroblast growth factor receptor; GLIPR1, GLI Pathogenesis-Related 1; GSEA, gene set enrichment analysis; Leprel1, leucine proline-enriched proteoglycan; NT shRNA, non-target shRNA; PBS, phosphate-buffered saline; PI, propidium iodide; PI3K, phosphatidylinositol-4, 5-bisphosphate 3-kinase; PRMT5, protein arginine methyltransferase 5; RT-PCR, reverse transcription polymerase chain reaction; SEM, the standard error of the mean; shRNA, small hairpin RNA; TGFβ, transforming growth factor beta; WDR77, WD repeat-containing protein 77

## Additional files

Additional file 1: Table S1. Primers used for RT-PCR (DOCX 20 kb) Additional file 2: Figure S1. Silencing p44 or PRMT5 expression did not significantly affect apoptosis. a A549 cells expressing NT shRNA, p44 shRNA or PRMT5 shRNA were submitted for TUNEL assay. b Percentage of TUNEL-positive cells in A549 cells infected with NT, PRMT5 or p44 shRNA-expressing lentivirus. (JPG 284 kb) Additional file 3: Figure S2. GSEA enrichment plot indicates that genes up regulated by PRMT5 shRNA were over-represented on the gene list, whose expression is negatively associated with cell proliferation. (JPG 970 kb) Additional file 4: Figure S3. GSEA enrichment plot indicates that genes up regulated by PRMT5 shRNA were over-represented on the gene list, whose expression is negatively associated with cell cycle progression. (JPG 974 kb) Additional file 5: Figure S4. Immunostaining of p44 in lung hyperplasia. P44-expressing cells are stained in brown. (JPG 1483 kb) Additional file 6: Figure S5. Immunostaining of PRMT5 in lung hyperplasia. PRMT5-expressing cells are stained in brown. (JPG 1464 kb) Additional file 7: Figure S6. Immunostaining of ErbB2, ErbB3 and FGFR3 in lung cancer samples. ErbB2-, ErbB3-, or FGFR3-expressing cells are stained in brown. (JPG 2494 kb) Additional file 8: Figure S7. The heatmap shows expression of PRMT5, WDR77 and their target genes in healthy normal and primary lung tumor tissues. The expression heatmap was created from the lung TCGA (*n* = 1.124) RNA-Seq gene expression data set (https://genome-cancer.ucsc.edu/download/public/xena/TCGA/TCGA.LUNG.sampleMap/HiSeqV2). (JPG 1076 kb)
