# Supplementary material for: Protein arginine methyltransferase 5 regulates multiple signaling pathways to promote lung cancer cell proliferation
Source: BMC Cancer. 2016 Aug 2;16:567. doi: 10.1186/s12885-016-2632-3 (PMC4970276; doi:10.1186/s12885-016-2632-3)
Supplement: Additional file 1: Table S1. — Primers used for RT-PCR (DOCX 20 kb) [file 12885_2016_2632_MOESM1_ESM.docx]

**sTable 1 Primers used for RT-PCR**

| Name | Sequence |
| --- | --- |
| GLIPR1-F | GACCCAGCACTAGCCCAAAT |
| GLIPR1-R | GTGATGGCGGAAGACACAGA |
| TIGAR-F  TIGAR-R  BTG2-F  BTG2-R  TP63-F  TP63-R  TMEM158-F  TMEM158-R  SPANXB2-F  SPANXB2-R  SDPR-F  SDPR-R  LAMC2-F  LAMC2-R  RAB3B-F  RAB3B-R  EFR3B-F  EFR3B-R  ARL14-F  ARL14-R  Leprel1-F  Leprel1-R  Ceacam1-F  Ceacam1-R  EMP1-F  EMP1-R  Dpysl4-F  Dpysl4-R  NMES1-F  NMES1-R  PTTG1-F  PTTG1-R | CTCTGACTGTTGTCCGGCAT  TGCATGGTCTGCTTTGTCCT  TGCATCCCTGGCTCCTCTAT  AGATTCCTGGCTGGAAAGCA  CGTTTCGTAGAAACCCCAGC  CTGCATGCGGATACAGTCCA  GGTGTGCTTCGTGCTGTAGT  GCAGGGGGATGCAATAGAGG  TGTGAATCCAACGAGGCCAA  GCGTGGTCATTCACCAGTTC  AACGCGGTTAGGAAGTGTGT  CAGGACTGGCAGAGGTTCAG  GGAGCTGGAGTTTGACACGA  GAGCATGGAGCTGGAAGGTT  AGATGCCGAACGTGTGATGT  AATGCCCCTTCTCTTGCTCC  CATCTCACCTGTGCCCTCTTT  AAGTCTCCATCCAGCTCCGT  ATCCGCAAACCAAACAAGCC  TCCACAACATACACCAGCCC  TGATGACTTTGAAGGAGGAGAA  AGAGCCACAGCACACCTCTT  CTCAGCCTCTCCTGCTATGC  TGTGGTCTTGCTGGCTTTGA  CTGGCTGTGCATTCTTGTGG  CCTCCCAGATTCAACGGCTT  AGAGGTGGGAGGATCGTGAA  TTGGTGCCCTGACAGAAGTC  TGGGAAACTGTGGACCCTAC  CAGAAGCATTTCCGCACACT  TGGTTGCTAAGGATGGGCTG  GTTTGAGGGGTCCCTTGGTC |
| ErbB3-F | GGTGCTGGGCTTGCTTTT |
| ErbB3-R | CGTGGCTGGAGTTGGTGTTA |
| FGFR1-F | CAAACCAAACCGTATGCCCG |
| FGFR1-R:  FGFR2-F: | AGGTGGCATAACGGACCTTG  CGCTGGGGAATATACGTGCT |
| FGFR2-R:  FGFR3-F  FGFR3-R | AGTCTGGCTTCTTGGTCGTG  AACAAGTTTGGCAGCATCCG  TCCTTGTCGGTGGTGTTAGC |
| FGFR4-F  FGFR4-R  NREP-F  NREP-R  SREBF1-F  SREBF1-R  AKTIP-F  AKTIP-R  ZWINT-F  ZWINT-R  PSMC3IP-F  PSMC3IP-R  NCAPG2-F  NCAPG2-R  ASGR1-F  ASGR1-R  FOXO3-F  FOXO3-R  mGLIPR1-F  mGLIPR1-R  mBTG2-F  mBTG2-R  mLeprel1-F  mLeprel1-R  mCeacam1-F  mCeacam1-R  mErbB3-F  mErbB3-R  mPTTG1-F  mPTTG1-R  mEMP1-F  mEMP1-R  mFGFR1-F  mFGFR1-R  mFGFR2-F  mFGFR2-R  mFGFR3-F  mFGFR3-R  mFGFR4-F  mFGFR4-R | GGAAGGCAGTTGGTGGGAAG  GCTACTGTCAGCTCCTGCTC  ACACGGTGGAAGGCTGATTC  TCCCTGGTACTTGGCTTTGC  CCCCACTTCATCAAGGCAGA  ATAGGCAGCTTCTCCGCATC  CATTGAAGGCACTGGGGTGA  CTGGTTTTCACGTCCCCTGT  TCTGCAGAGGGTAAGCTGTTG  TTGGGAGGTGAGGGAAGTCA  TGAGGAGGTCGGTTGAGTGG  ACTGGTCCTGATCCGCAAAAT  CCGAGCCCAGGGTTACAAAA  AGGCTGAAAGGATCAGAGGC  GTGGTTGTCTGTGTGATCGG  AGCTGGGACTCTAGCGACTT  GACCCCAGCACCAAGTCTAC  TGGCAATGAGTGGAGAGCTG  CCTCAAGCTGAAGTAGGCTGT  AGAGCTGCGGGGTTCTGATA  GCTCCAGGGAACTCCAAGTC  AACCTACCAGGGCATCAGGA  CCAACGCAGAGTTCCCTACAA  TAGCTTTCCAGGTGTGGCTTG  GCATACAGCGGCAGAGAGAT  TGTTGCTTGTGATGTTGGGC  TAAACGGGCTGAGTGTGACC  TCGGAGGTTAGGTAAGGGCA  TGGCGCAGTCTTCGAGTAAT  AGGCTTTTCGGCAACTCTGT  CCCTGTCCTACGGCAATGAA  TTCGCTGTGGGCGTAATGAT  GCCTGAACAAGATGCACTCC  CTCGACGGGCACTTGAACTT  AACCAGAAGAGCCACCAACC  CTGTCTACCGTCCTAGCTGC  GTCTCCACAGAGGCGTTCTC  CTCCTGCTGGCTAGGTTCAG  CTCCGGGACTAGCTGCAAAA  AGAGCTGATGCCCCTTTCAC |
