# Supplementary figures and images for: Protein arginine methyltransferase 5 regulates multiple signaling pathways to promote lung cancer cell proliferation
Source: BMC Cancer. 2016 Aug 2;16:567. doi: 10.1186/s12885-016-2632-3 (PMC4970276; doi:10.1186/s12885-016-2632-3)

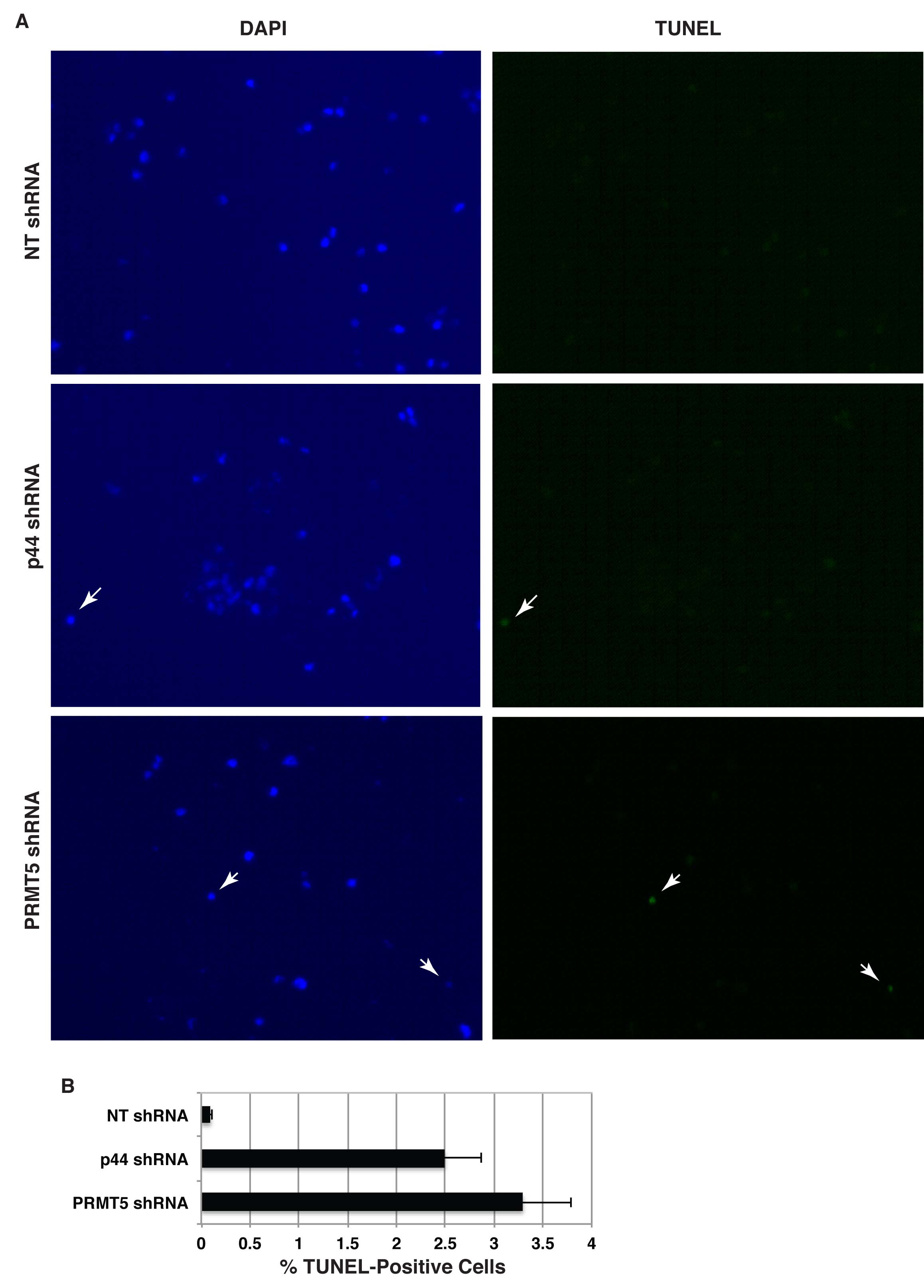

Supplement: Additional file 2: Figure S1. — Silencing p44 or PRMT5 expression did not significantly affect apoptosis. a A549 cells expressing NT shRNA, p44 shRNA or PRMT5 shRNA were submitted for TUNEL assay. b Percentage of TUNEL-positive cells in A549 cells infected with NT, PRMT5 or p44 shRNA-expressing lentivirus. (JPG 284 kb) [file 12885_2016_2632_MOESM2_ESM.jpg]

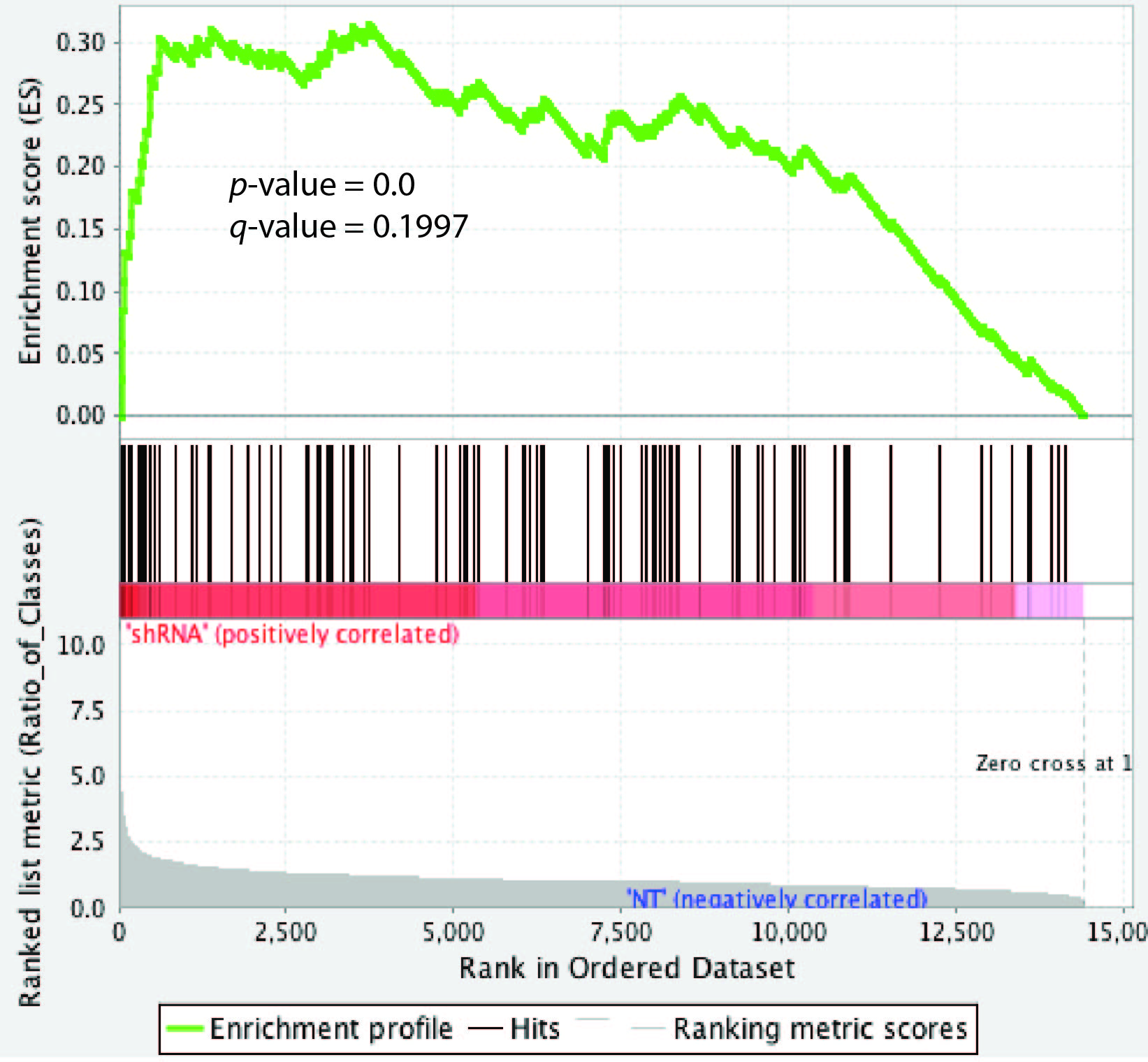

Supplement: Additional file 3: Figure S2. — GSEA enrichment plot indicates that genes up regulated by PRMT5 shRNA were over-represented on the gene list, whose expression is negatively associated with cell proliferation. (JPG 970 kb) [file 12885_2016_2632_MOESM3_ESM.jpg]

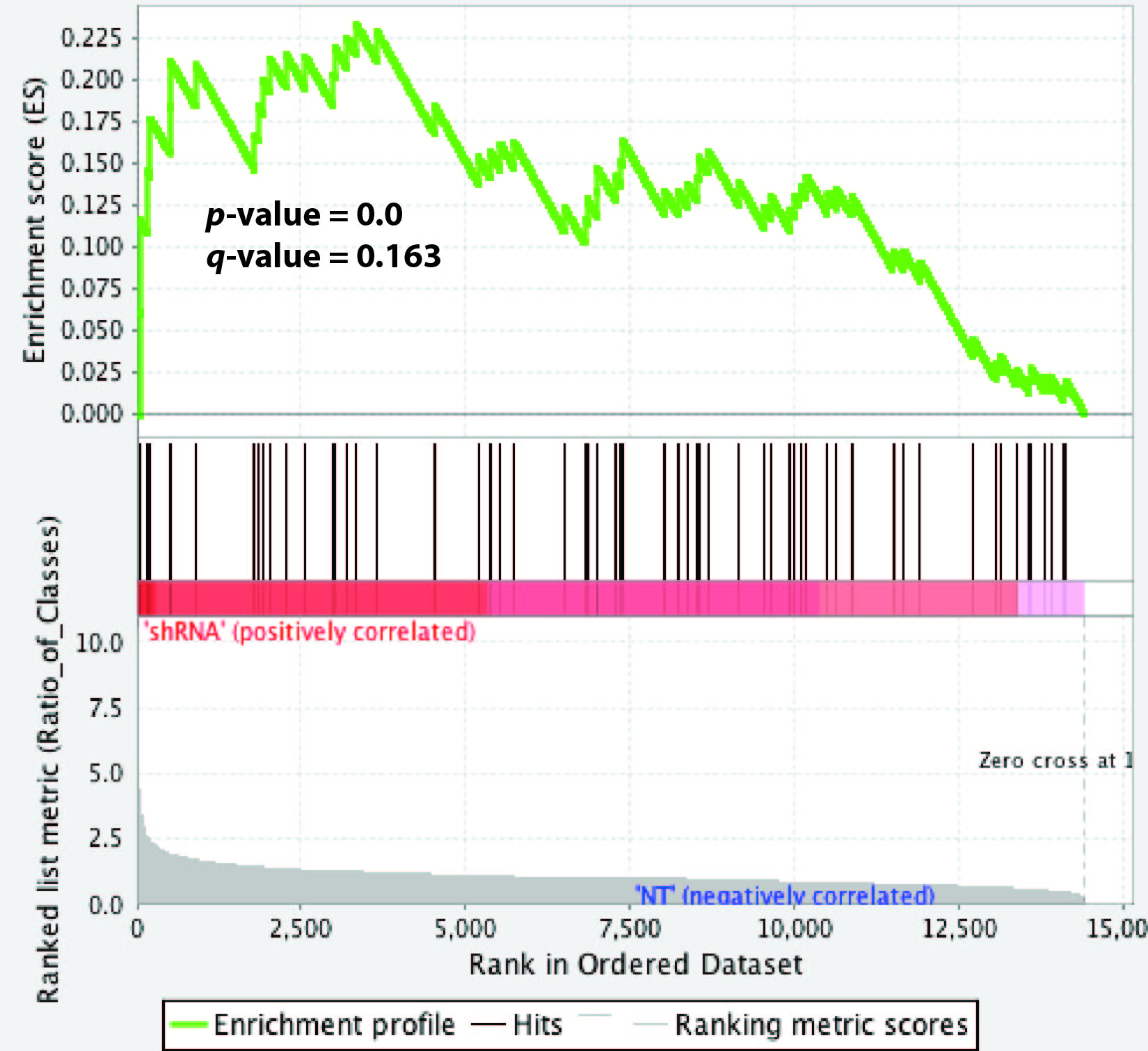

Supplement: Additional file 4: Figure S3. — GSEA enrichment plot indicates that genes up regulated by PRMT5 shRNA were over-represented on the gene list, whose expression is negatively associated with cell cycle progression. (JPG 974 kb) [file 12885_2016_2632_MOESM4_ESM.jpg]

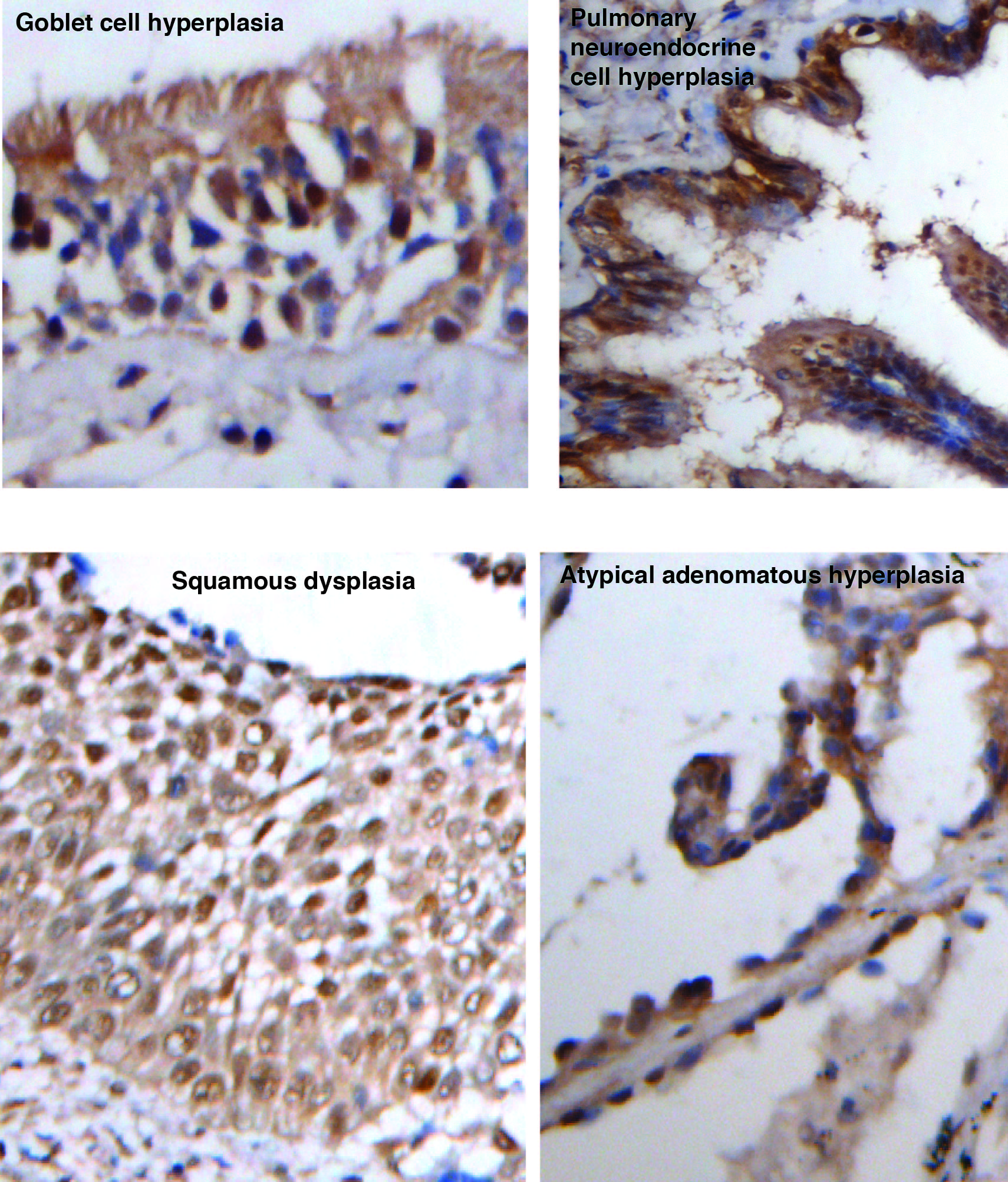

Supplement: Additional file 5: Figure S4. — Immunostaining of p44 in lung hyperplasia. P44-expressing cells are stained in brown. (JPG 1483 kb) [file 12885_2016_2632_MOESM5_ESM.jpg]

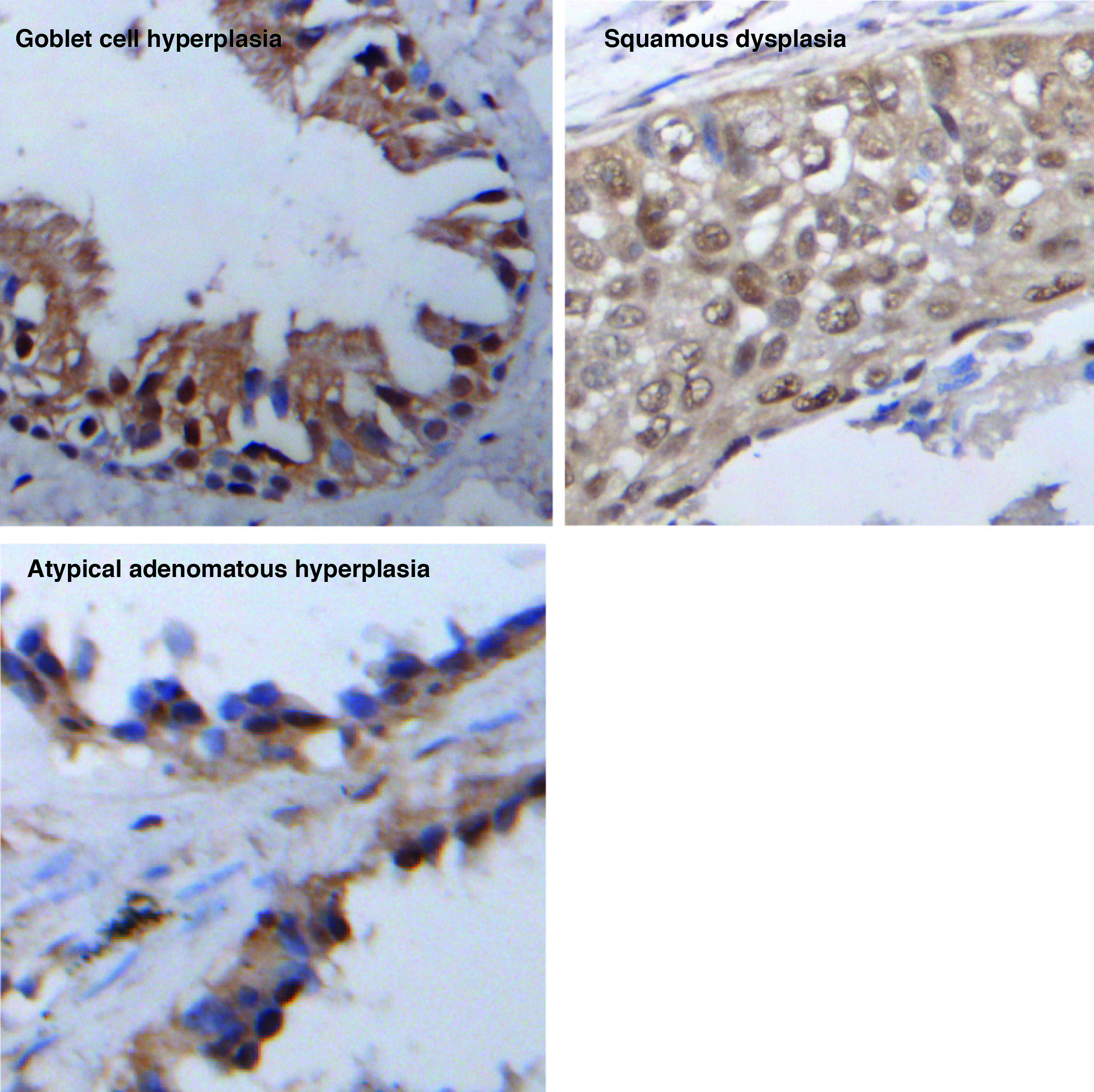

Supplement: Additional file 6: Figure S5. — Immunostaining of PRMT5 in lung hyperplasia. PRMT5-expressing cells are stained in brown. (JPG 1464 kb) [file 12885_2016_2632_MOESM6_ESM.jpg]

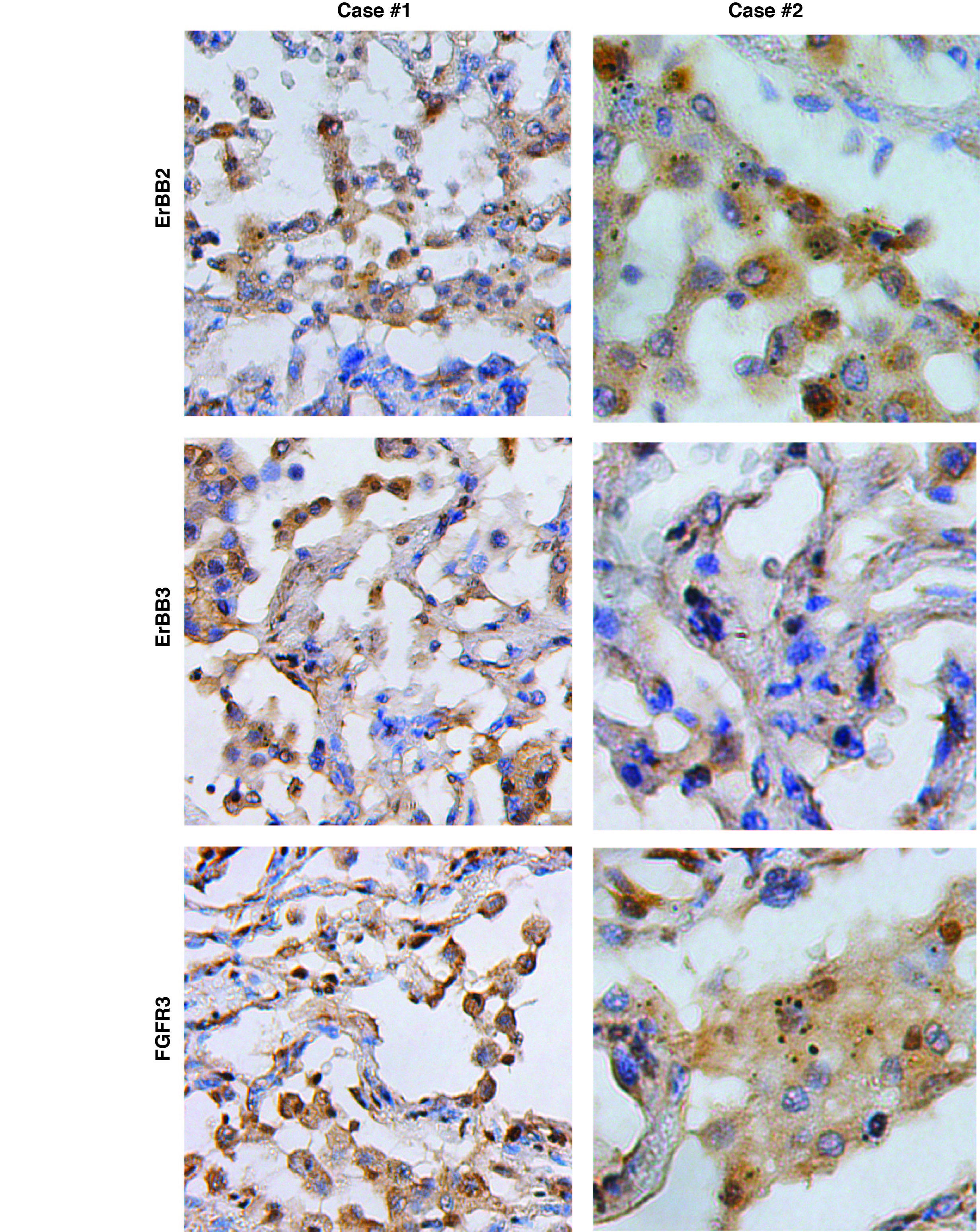

Supplement: Additional file 7: Figure S6. — Immunostaining of ErbB2, ErbB3 and FGFR3 in lung cancer samples. ErbB2-, ErbB3-, or FGFR3-expressing cells are stained in brown. (JPG 2494 kb) [file 12885_2016_2632_MOESM7_ESM.jpg]

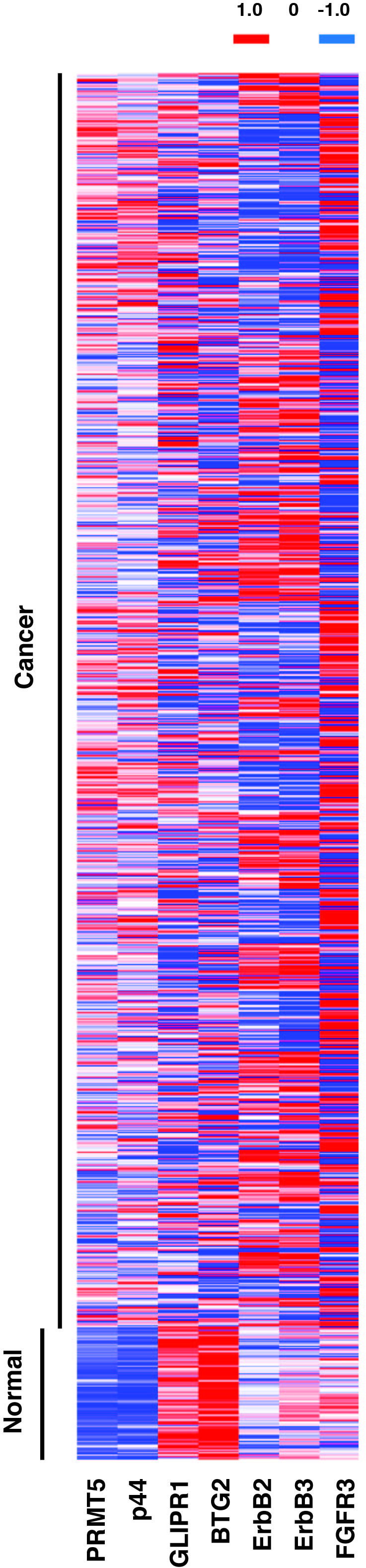

Supplement: Additional file 8: Figure S7. — The heatmap shows expression of PRMT5, WDR77 and their target genes in healthy normal and primary lung tumor tissues. The expression heatmap was created from the lung TCGA (n = 1.124) RNA-Seq gene expression data set (https://genome-cancer.ucsc.edu/download/public/xena/TCGA/TCGA.LUNG.sampleMap/HiSeqV2). (JPG 1076 kb) [file 12885_2016_2632_MOESM8_ESM.jpg]
